# Complicated third sternotomy for left ventricular outflow tract obstruction secondary to mitral valve prosthesis strut

**DOI:** 10.21542/gcsp.2026.13

**Published:** 2026-04-30

**Authors:** Rishab Agarwal, AlleaBelle Bradshaw, Michelle Carvajal, Jennifer S. Lawton

**Affiliations:** 1 Eastern Virginia Medical School, Norfolk, VA; 2 Division of Cardiac Surgery, Department of Surgery, Johns Hopkins University School of Medicine, Baltimore, MD

## Abstract

**Introduction**: LVOT obstruction after surgical bioprosthetic MVP placement is uncommon and the influence of the MVP strut width has seldom been examined.

**Case presentation**: A 74-year-old female presented with palpitations, chest pain, and shortness of breath. She had MVR with 31-mm bioprosthetic valve 2 years prior. TEE confirmed LVOT obstruction secondary to a MVP strut, causing a peak gradient of 55 mmHg. The redo operation was complicated by iatrogenic Type A aortic dissection. The patient was immediately cooled for DHCA with RCP and ascending aortic replacement was performed. MVR was then performed with a prosthesis with narrower strut width. Recovery after surgery was uneventful and post-bypass TEE confirmed absence of LVOT obstruction (gradient of 4 mmHg).

**Discussion**: This case is of interest for two reasons. Primarily, the overall profile of the valve, including the width of the struts may play a significant role in the development of LVOT obstruction after MVR. Additionally, the patient had an ITAAD requiring prompt and thoughtful management.

**Conclusions**: This case underscores the importance of evaluating MVP profile in relation to patient anatomy. Furthermore, intraoperative Type A aortic dissection is a rare but lethal phenomenon requiring swift decision-making and technical ability, and both are important considerations for trainees.

## Introduction

Left ventricular outflow tract (LVOT) obstruction secondary to surgical placement of a bioprosthetic mitral valve prosthesis (MVP) has an estimated incidence less than 1%^1^. This phenomenon results from a complex interplay of mechanisms, including improper positioning of the MVP, a potentially preserved anterior leaflet, septal hypertrophy, a small ventricular cavity, and/or subsequent pannus formation^1^. An easily modifiable factor is the choice of MVP, as LVOT obstruction is more likely with the insertion of small bioprosthetic valves with tall struts which decrease the aorto-mitral angle and protrude into the LVOT^2,3^. However, strut width is seldom considered and inconsistently reported by manufacturers, although it may also contribute^4,5^.

**Figure 1.**
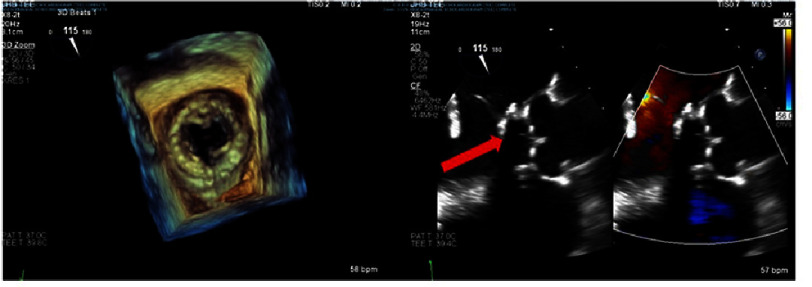
Preoperative transesophageal echocardiography. Left: Three-dimensional reconstruction of the previously placed, normally functioning bioprosthetic mitral valve with LVOT obstruction caused by a strut. Right: Mid-esophageal aortic long-axis view demonstrates obstruction of the LVOT by a strut (arrow). LVOT; left ventricular outflow tract.

A case of mitral valve replacement (MVR) for LVOT obstruction from a prior MVP, complicated by an iatrogenic intraoperative Type A aortic dissection (ITAAD) is presented. We emphasize that the overall profile of the valve, including the width of the struts may play a significant role in the development of LVOT obstruction after MVR.

## Case

A 74-year-old female presented with palpitations, chest pain, and shortness of breath. Five years prior, she had a mitral and tricuspid valve repair and left atrial appendage ligation for endocarditis. Two years prior, she had MVR with a 31 mm Epic (Abbott, Abbott Park, IL) bioprosthetic valve for symptomatic mitral regurgitation.

A transthoracic echocardiogram showed an ejection fraction of 55%, normally functioning bioprosthetic MVR, and an increased LVOT gradient of 37 mmHg due to strut impingement. LVOT diameter was 1.85 cm and interventricular septum diastolic thickness was 0.59 cm. Left ventricular diastolic and systolic diameters were 5.79 and 3.65 cm respectively. The anterior mitral leaflet was preserved at the time of the second operation (MVR). Transesophageal echocardiogram (TEE) confirmed a well-seated valve with mobile leaflets, no paravalvular leak, and a mean mitral gradient of 4.6 mmHg (Figure 1). However, a strut narrowed the LVOT, causing a peak gradient of 55 mmHg. Cardiac catheterization revealed dynamic LVOT obstruction (peak-to-peak gradient 50 mmHg, mean 40 mmHg). Due to her symptoms, it was recommended to proceed with redo MVR to address the primary cause of her LVOT obstruction.

After uneventful redo sternotomy, the aorta and right atrium were cannulated for cardiopulmonary bypass. A femoral arterial line was placed. The aortic cross-clamp was placed, and hypothermic, hyperkalemic blood (4:1) cardioplegia was delivered via the aortic root. The heart did not arrest immediately, and bleeding was noted from behind the cross clamp, thought to be the pulmonary artery (due to dense adhesions). The patient was immediately cooled for deep hypothermic circulatory arrest (DHCA). Pump suckers were utilized. The superior vena cava was cannulated for retrograde cerebral perfusion (RCP).

The patient was cooled to 18 ° C for DHCA. The cross-clamp was removed, and there was partial transection of the aorta where the cross-clamp had been. There were no other injuries. After aortotomy, direct cannulation of both coronaries was utilized for cardioplegia delivery. The Epic mitral valve strut was visualized beneath the aortic valve leaflets, clearly obstructing the LVOT. The dissected aorta was resected. A 30 mm Hemashield graft was anastomosed to the distal ascending aorta, cardiopulmonary bypass was resumed via the graft, and the patient was rewarmed. The proximal graft anastomosis to the aorta was performed.

Attention was then turned to the mitral valve via Waterston’s groove. This area was densely scarred, limiting visibility during removal of the old prosthesis. A prosthesis with a lower profile strut (Magna Ease, Edwards LifeSciences, Irvine, CA) was chosen and care was made to ensure no strut was in the LVOT. The patient was weaned from cardiopulmonary bypass on minimal support and recovery after surgery was uneventful. Post-bypass TEE confirmed absence of LVOT obstruction (gradient of four mmHg).

The patient was followed for 11 months postoperatively. She was asymptomatic and she completed three months of cardiac rehab and resumed work. Her echocardiogram revealed a mean mitral valve gradient of seven mm Hg across the Magna Ease valve.

## Discussion

This case of a complicated 3rd sternotomy and mitral valve operation is of interest for two reasons. Primarily, the overall profile of the valve, including the width of the struts may play a significant role in the development of LVOT obstruction after MVR. Additionally, the patient had an ITAAD requiring prompt and thoughtful management.

**Figure 2.**
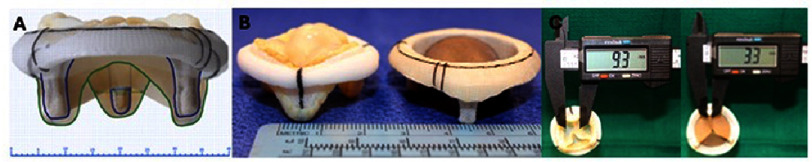
Comparison of bioprosthetic mitral valve strut profiles. (A) Overlay of the Epic™ mitral valve (Abbott; green outline) and the Magna Ease™ mitral valve (Edwards Lifesciences; blue outline). The Epic valve (29 mm size) demonstrates a taller total profile height of approximately 19 mm^6^. In contrast, the Magna Ease valve (29 mm size) exhibits a lower anterior profile of approximately eight mm, and a total profile height of 17 mm, offering a lower-profile design^7^. B: Side-by-side comparison of the 29 mm size Epic mitral valve (left) and the 29 mm size Magna Ease mitral valve (right). C: Direct caliper measurments of the strut width at the midpoint are 9.3 mm in the Epic valve (left) compared to 3.3 mm in the Magna Ease valve (right). Furthermore, the strut of the Epic valve appears to be wider overall and gradually tapers, while the Magna Ease valve is narrow throughout. These quantitative differences underscore the need for careful consideration of valve choice during mitral valve replacement.

Strut width is often underreported and may play a significant role in the development of LVOT after MVR^4,5^. Figure 2 shows an overlay and side-by-side comparison of the Abott Epic™ Plus valve and the equivalent sized Edwards Magna Ease™ mitral valve. Compared to the 29 mm size Magna Ease valve, the 29 mm size Epic valve demonstrates a taller total profile height (19 vs 17 mm)^6,7^. Importantly, the struts of the Epic valve appear to be wider and gradually taper, while the Magna Ease struts are narrow throughout. Direct caliper measurements of the strut widths at the midpoint are 9.3 mm in the 29 mm sized Epic valve compared to 3.3 mm in the 29 mm Magna Ease valve. These quantitative differences underscore the need for careful consideration of valve choice during MVR. To support this decision-making process, manufacturers can improve their reporting standards for prosthesis dimensions to include strut width which may aid in assessing and preventing LVOT obstruction^4^. Surgeons may also consider using preoperative CT imaging and 3D echocardiography to help assess LVOT anatomy and predict LVOT obstruction (well studied strategies in transcatheter MVR planning^8,9^). This may prove especially helpful for patients who exhibit risk factors for post-operative LVOT obstruction.

In addition to considering the valve profile, surgeons must take precautions to prevent LVOT obstruction during placement of the valve. Prosthetic valves are designed with marks on the sewing ring to help guide positioning. Proper placement requires a thorough understanding of the spatial relationship between the aortic valve, mitral valve, and LVOT.

This case emphasizes effective management of ITAAD. While the incidence of ITAAD is less than 1%, mortality can approach 50%^10^. This patient’s dissection was successfully managed with immediate DHCA with RCP and replacement of the ascending aorta. Importantly, immediate neuroprotective interventions including hypothermia were quickly implemented when the dissection was identified.

### What have we learned?

**Figure 3.**
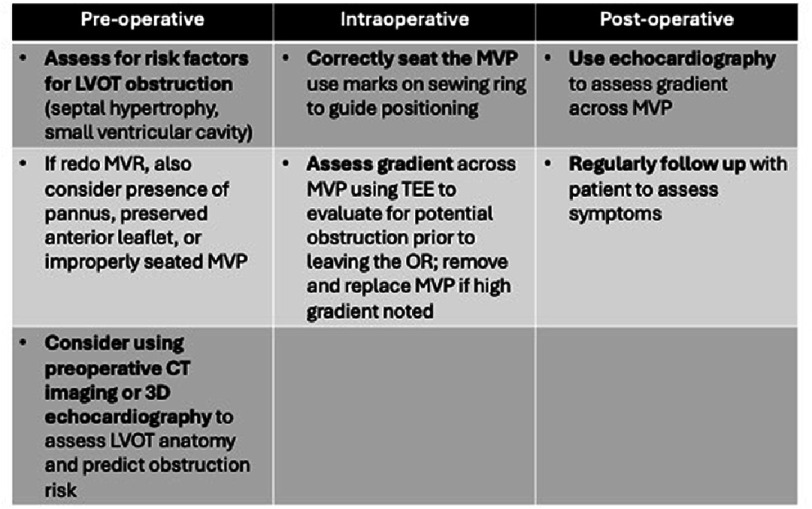
Decision making framework and important considerations for trainees to prevent LVOT obstruction in MVR. The framework has separated into pre-operative, intraoperative, and post-operative stages to help organize important considerations. CT, computed tomography; LVOT, left ventricular outflow tract; MVP, mitral valve prosthesis; MVR, mitral valve replacement; OR, operating room; TEE, transesophageal echocardiography.

This case underscores the importance of evaluating MVP profile in relation to patient anatomy. MVP strut width may play a significant role in the pathogenesis of LVOT obstruction after MVR and should be considered as a contributing factor. Efforts should be made by manufactures to report this measurement. Furthermore, ITAAD is a rare but lethal phenomenon requiring swift decision-making and technical ability; successful management involves immediate neuroprotective interventions once dissection is identified. Overall, the appropriate selection of prosthesis and swift management of intraoperative complications are important considerations for trainees. A decision making framework to prevent LVOT obstruction in MVR has been highlighted in Figure 3.

## Author statement

**Conceptualization:** R.A., A.B., and J.S.L.

**Data curation:** A.B. and M.C.

**Investigation:** R.A.

**Supervision:** J.S.L.

**Writing - original draft:** R.A. and A.B.

**Writing - review & editing:** R.A., A.B., M.C., and J.S.L.
